# Tell Me Where You’ve Been and I’ll Tell You How You’ll Evolve

**DOI:** 10.1128/mBio.02043-20

**Published:** 2020-10-27

**Authors:** Marco Fumasoni

**Affiliations:** a Department of Molecular and Cellular Biology, Harvard University, Cambridge, Massachusetts, USA; University of Pittsburgh

**Keywords:** *Desulfovibrio vulgaris*, experimental evolution, genetic divergence

## Abstract

The reproducibility of adaptive evolution is a long-standing debate in evolutionary biology. Kempher et al. (M. L. Kempher, X. Tao, R. Song, B. Wu, et al., mBio 11:e00569-20, 2020, https://doi.org/10.1128/mBio.00569-20) used experimental evolution to investigate the effect of previous evolutionary trajectories on the ability of microbial populations to adapt to high temperatures. Despite the divergence caused by adaptation to previous environments, all populations reproducibly converged on similar final levels of fitness. Nevertheless, the genetic basis of adaptation depended on past selection experiments, reinforcing the idea that previous adaptation can dictate the trajectories of later evolutionary processes.

## Abstract (precis)

The reproducibility of adaptive evolution is a long-standing debate in evolutionary biology. Kempher et al. (M. L. Kempher, X. Tao, R. Song, B. Wu, et al., mBio 11:e00569-20, 2020, https://doi.org/10.1128/mBio.00569-20) used experimental evolution to investigate the effect of previous evolutionary trajectories on the ability of microbial populations to adapt to high temperatures. Despite the divergence caused by adaptation to previous environments, all populations reproducibly converged on similar final levels of fitness.

## INTRODUCTION

Since the formulation of the theory of evolution by natural selection by Darwin and Wallace (1) and its integration with genetics during the modern synthesis (2), natural selection has been recognized as the major force shaping the features of living beings. One of the most striking examples of the effects of this force is convergent evolution: the independent acquisition of similar traits by organisms subjected to the same environment. If the selective force determines the outcome of evolution, we expect some sort of “determinism”: similar outcomes in response to the same selective force, something that, to a large extent, has been observed in nature (3). The paleontologist Stephen J. Gould criticized the deterministic view, claiming that historical events play a major role in the outcome of evolutionary processes, a concept broadly defined as “contingency” (4). The critique was summarized by Gould in his analogy of “replaying the tape of life”, which proposed that the outcome of replaying the history of life on earth would lead to a remarkably different outcome from what we currently observe. Unfortunately, this thought experiment is not practically feasible, and thus the critique has sparked a long-standing debate on the relative contributions of selective forces and historical contingency in determining the genetic and physiological trajectories of adaptive evolution.

Microorganisms offer a unique opportunity to investigate the reproducibility of evolutionary processes by allowing scientists to study several parallel populations, for many generations and under the same controlled environmental conditions (5). Many microbial evolution experiments have been conducted to date, and while parallel evolution of replicate populations has been extensively observed, many of these experiments have been conducted starting with multiple populations of genetically identical organisms. However, isolated groups of organisms in nature are often subjected to different environments, and the effect of the genetic and phenotypic divergence that these environments generate on the adaptation to the same selective force is not well understood. A specific type of microbial experiment has been designed to investigate this question, by preexposing organisms to environments that mimic the different historical contingencies encountered by populations in nature. Although limited in number, and in the breath of species and conditions tested, studies based on these experiments have provided solid evidence for the phenotypic convergence of previously diversified populations (6, 7) but have sometimes reported differences in how it is achieved at the genetic level (8, 9). A clear picture that portrays the evolution of organisms under such conditions is thus yet to emerge.

**FIG 1:**
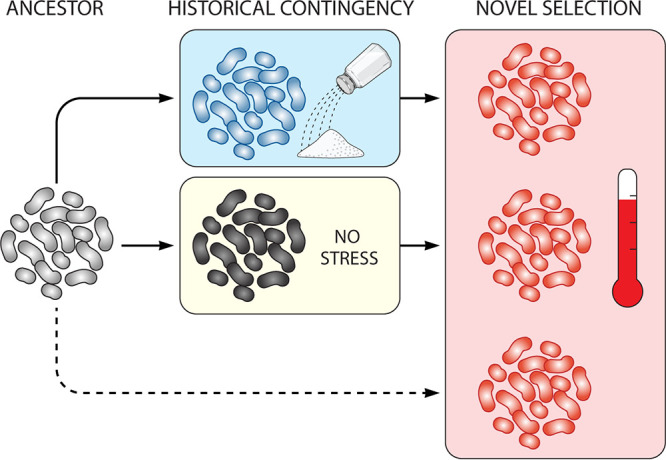
Experimental design schematic. Desulfovibrio vulgaris Hildenborough ancestor cells were first evolved in the presence of a high salt concentration (light blue box) or in the absence of stress (light yellow box). This stage mimics the historical contingency of adaptation of organisms to different environments in nature. Subsequently, the strains that had been evolved under both sets of conditions, together with the original ancestors that generated them, were subjected to the same novel selection condition: high temperature (red box). Dashed lines represent cryogenic preservation of cells until the novel selection was applied.

Kempher et al. (10) set out to address the effect of previous evolutionary adaptations on the response to a novel selection by using the obligate anaerobic bacterium Desulfovibrio vulgaris Hildenborough, a model organism for studying the energy metabolism of sulfate-reducing bacteria. First, they subjected the same strain of ancestor cells to either of two different environments, high salt concentrations or standard laboratory conditions, and evolved cells under these conditions. This phase created historical contingency by subjecting individuals of the same genotype to different environments that could influence their future adaptation. As a consequence of the selective pressures, the two populations differentiated phenotypically and genotypically. The authors then asked how these differences influenced their future adaptation to a common, novel selection, high temperature. They compared three populations: cells adapted to high salt, cells adapted to standard medium, and cells that had no prior adaptation. This experiment differs from previous ones in two ways: it uses a less common model organism and the historical conditions applied before the common selection differ more dramatically than those of previous studies (6). The authors found that while the phenotypes of the three different groups of populations converged to similar fitness levels, the underlying genetic changes differed depending on the environment in which the strains had previously evolved. Only three genes acquired mutations in at least one population in all the three groups, reflecting limited genetic convergence among populations previously evolved under different conditions. Parallelism among populations belonging to the same group was instead more marked. For instance, the *hspC* gene, which encodes a heat shock protein, was mutated in all six populations evolved in the absence of stress (Fig. 1, light yellow box) but not in any of the other populations analyzed. Overall, the study contributed further evidence that phenotypic convergence can be achieved through diverse genetic changes. Furthermore, despite the differences in the model organism and in the type of selective pressures applied, Kempher et al. support a general trend observed in previous studies where natural selection drives convergence at the phenotypic level but historical contingency produces differences in the details of how genotypes achieve the phenotypes (11).

Outside this general trend, a few studies have reported a more dramatic effect of historical contingency on the adaptation to a common environment. In one study, Escherichia coli populations previously evolved in different media showed significantly different fitness results after adaptation to a common medium supplement with glycerol (12). In another study, two populations of bacteriophage ϕ6 were first allowed to independently compensate for the same detrimental mutation under two different conditions. The resulting populations were then further propagated in the same environment, and while one increased in fitness, the other did not (13). Why do studies using similar experimental designs show different levels of phenotypic variability between evolved populations? Despite the common experimental strategy, there are still many variables at play. One likely factor is the extent to which the populations subjected to the same selection are phenotypically and genotypically divergent; comparative biologists report that, outside the lab, closely related species adapt in more-similar ways than distantly related taxa (11). Another possibly related factor influencing the outcome could be the extent to which the genetic targets of the current selection overlap or interact with those that mutated in response to previous selective pressure; even when the previous selection is strong, adaptation may involve mutations in processes that are not relevant to the adaptation to the novel selection and that have little or no influence on it. Alternatively, even a mild past selection could modify genes that are pivotal for future responses to selection, to the point of preventing adaptation or dramatically changing its outcome.

I believe that the answer to these questions is less likely to come from a single, albeit well-designed experiment than it is to emerge from progressively adding key and diverse sets of observations and integrating them to produce a clearer picture. In this context, works like that by Kempher et al. make the important contribution of using different organisms and selective pressures, adding breadth to the observations on experimental evolution. More in-depth studies will likely be required to investigate the molecular and cellular bases of adaptation. By which mechanisms are organisms adapting to the selections applied? How do preexisting mutations prevent or favor specific adaptive solutions? Answering these questions, by analyzing and reconstructing evolutionary trajectories, will require a combination of performing studies in organisms with great genetic tractability (14) and increasing the tractability of a greater variety of experimental organisms.

Any resolution of the debate concerning the roles of natural selection and historical contingency in evolution lies well into the future, and a general, quantitative solution to the balance between these two forces may not even exist. Nevertheless, further studies adding breadth and depth to experimental evolution will expand our understanding of how evolution works and will increase our ability to predict the outcome of evolutionary processes.
